# Osteochondritis dissecans of the elbow: excellent mid-term follow-up results in teenage athletes treated by arthroscopic debridement and microfracture

**DOI:** 10.3325/cmj.2012.53.40

**Published:** 2012-02

**Authors:** Ivan Bojanić, Tomislav Smoljanović, Stjepan Dokuzović

**Affiliations:** 1 Department of Orthopedic Surgery, University Hospital Center Zagreb, Zagreb, Croatia; 2 School of Medicine, University of Zagreb, Zagreb, Croatia.; 3 Department of Orthopedic Surgery, University Hospital Dubrava, Zagreb, Croatia

## Abstract

**Aim:**

To extend the microfracture procedure, which has been proven successful on osteochondritis dissecans (OCD) lesions in the knee and ankle, to OCD lesions in the elbow.

**Methods:**

Nine young patients were treated by arthroscopic debridement and microfracture by a single surgeon. The average age at operation was 15.0 years (median 15; range 12-19). The average length of the follow-up was 5.3 years (median 5; range 2-9). The follow-up included physical examination and patient interview with elbow function scoring. Success of treatment was determined according to pre-operative and follow-up Mayo Elbow Performance Index scores and the patients’ return to sports.

**Results:**

Eight patients scored excellent results on the follow-up and 1 scored a good result. Four out of 9 patients were able to increase their training intensity, 2 returned to the same level of activity, 2 changed sports (due to reasons unrelated to the health of their elbow), and 1 left professional sports and started training only recreationally. No patients stopped participating in sports altogether.

**Conclusions:**

We advocate arthroscopic microfracturing, followed by a strict rehabilitation regime, as a highly effective treatment for OCD of the humeral capitellum.

Osteochondritis dissecans (OCD) represents a transchondral fracture, with separation of the articular cartilage from subchondral bone, resulting from repeated trauma and overuse (1,2). The precise incidence and prevalence of OCD of the elbow are still unknown, except to say that they are highest among pre-adolescent and adolescent athletes, particularly those involved in throwing sports (1,3-6). The most frequent site in the elbow is the humeral capitellum, though it has been described in the trochlea, olecranon, and radial head (7-9).

OCD of the elbow can cause permanent elbow disability in adolescent athletes if left untreated. There is an overall consensus as to when surgery is indicated, though the gold standard is still debatable. Our aim is to extend the use of the microfracture procedure, which has proven successful on OCD lesions in the knee and ankle (10-14), to OCD lesions in the elbow.

## Materials and methods

Between 2002 and 2009, the first author (I. B.) performed 9 arthroscopic elbow surgeries on patients between the ages of 12 and 19 (average 15.0 years; median 15.0 years) by performing debridement and microfracturing. The 3 youngest patients (all aged 12 years) still had their capitellar physis open on radiographs. All patients had radiographically evident type II and type III (radiographic typing according to Bradley et al) (15). OCD lesions of the humeral capitellum were referred to the senior author for surgical treatment after failed attempts at conservative treatment in other institutions. Six were men and 3 were women, all were involved in sports and in 5 of 9 cases (55.5%) the dominant arm was affected. Clinically, all patients experienced medium to severe pain, some with instability and profoundly limited range of motion (Table 1). Arthroscopic grading of lesions was done using the classification proposed by Baumgarten et al (16).

**Table 1:** Characteristics of the study participants. All patients were treated by the same surgeon and by the same technique – arthroscopic debridement with microfracture*

Sex/age at operation	Arm affected/ dominant arm	Duration of symptoms (months)	Chief complaints	MEPI before/after operation	Radiographic defect type^†^	Grade of lesion^‡^	Sport	Level after operation	Follow-up (years)
M/16	L/R	18	severe pain, ROM	35/100	III	5	gymnastics (competitive)	improved	9
F/12	L/R	6	severe pain, loss of function	15/100	III	5	gymnastics (competitive – national team)	same for 2 y, then decreased§	7.5
F/12	R/R	33	moderate pain	70/100	II	3	gymnastics (competitive – national team)	same for 4 y, then decreased§	7.5
M/19	R/R	80	severe pain	55/100	II	4	basketball (recreational)	changed to kickboxing	6
M/15	R/R	24	moderate pain	70/100	II	4	track & field throwing sports (recreational)	changed to weight training	5
M/14	R/L	4	moderate pain	70/85	II	3	handball (competitive)	dropped to recreational	4.5
M/18	R/R	4	severe pain	55/100	II	4	waterpolo (competitive - national team)	improved	4
M/17	R/R	24	severe pain	55/100	II	3	tennis (competitive)	improved	2.5
F/12	R/L	24	severe pain	55/100	II	4	gymnastics (competitive – national team)	improved	2

*Abbreviations: M – male; F – female; L – left; R – right; ROM – range of motion; MEPI – Mayo Elbow Performance Index.

†Radiographic type according to Bradley et al. Ia – almost normal x-ray with low signal MRI; Ib – capitellar rarefactions, flattening, and/or sclerosis on x-ray, subchondral cysts and fluid on MRI; II – sclerotic margin around a well defined undisplaced fragment on x-ray; III – chronic lesions with loose bodies on x-ray and MRI; IV – associated radial head osteochondritis dissecans (18).

‡Arthroscopic grading system according to Baumgarten (15). Grade 1 lesions have smooth, soft, ballotable articular cartilage. Grade 2 lesions have cartilage fibrillations or fissuring. Grade 3 lesions have exposed bone with a stable osteochondral fragment. Grade 4 lesions have a loose but nondisplaced fragment. Grade 5 lesions have a displaced fragment with resultant loose bodies.

§These patients decreased their pre-operative sporting activity due to reasons unrelated to the status of their elbows.

**Figure 1:**
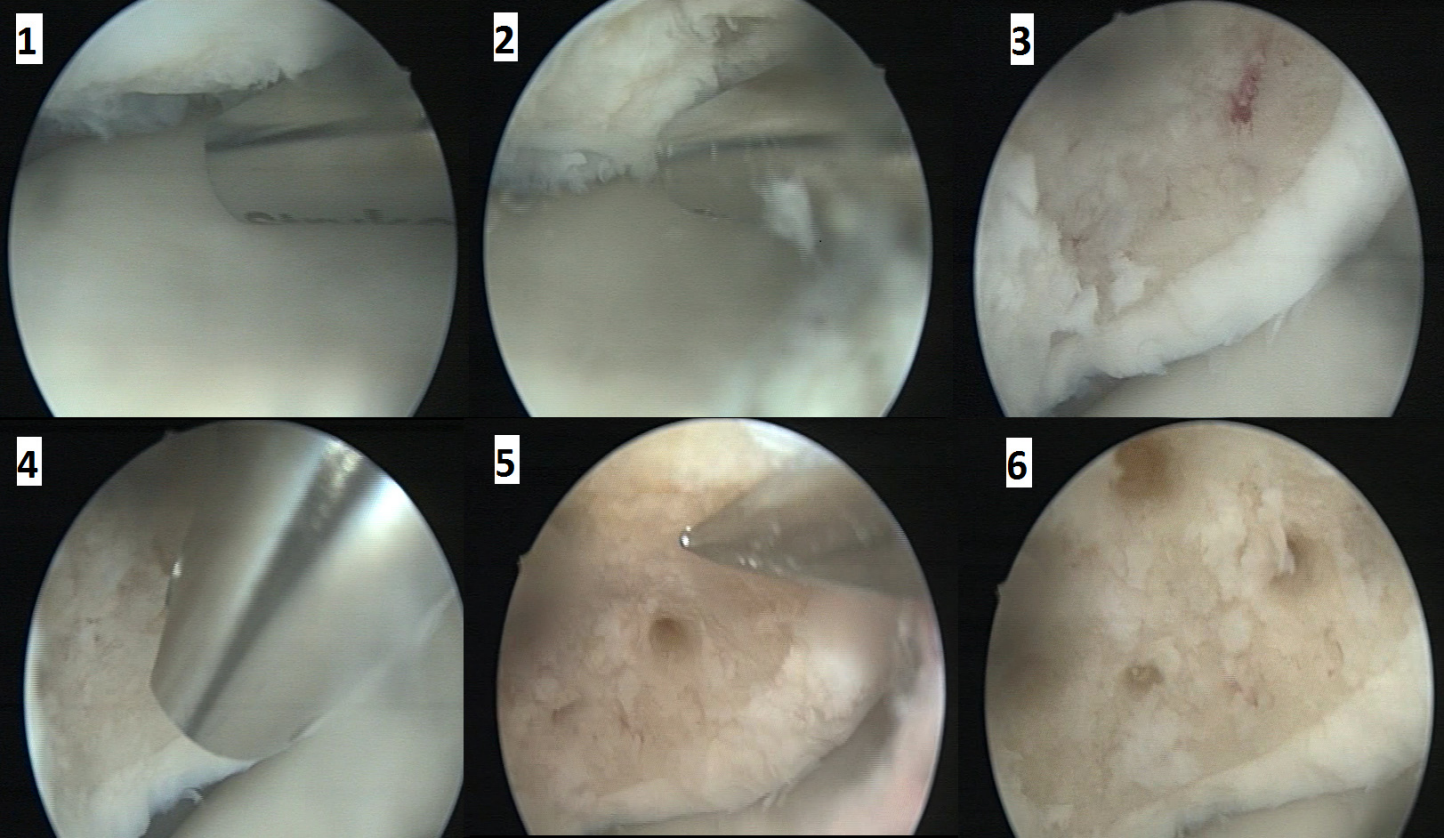
Sequential views of a typical arthroscopic debridement and microfracture procedure. This is the right (dominant) elbow in a 17-year old male tennis player whose chief complaint was pain. 1 – osteochondral lesion visualized through direct lateral portal with the shaver in the adjacent direct lateral portal; 2 – debridement of osteochondral lesion with shaver; 3 – debrided and cleaned defect with neatly modeled border at the interface with the surrounding healthy cartilage; 4 – removal of calcified cartilage from base of defect using shaver; 5 – piercing the subchondral bone using a microfracture awl; 6 – the lesion site after completion of procedure, the holes being 2-4 mm deep and 3 mm apart.

Elbow arthroscopy was performed in general anesthesia, using tourniquet control with the patient on a chest roll in the prone position. The arm was supported in a holder with the elbow in 90° of flexion. We used a standard 4.0-mm 30° arthroscope and began with creation of the proximal anteromedial portal, followed by creation of the proximal anterolateral portal. The anterior compartment was inspected and the cartilage evaluated for potential softening or fragmentation. If present, loose bodies were removed as well (they were present in 2 patients). The olecranon fossa was also inspected in the patients with radiographically evident or suspected loose bodies (through the straight posterior and posterolateral portals – this was done in 3 patients, followed by the posterior compartment which would be entered through a direct-lateral portal placed in the posterior soft spot in line with the lateral epicondylar ridge) and examined. An adjacent direct-lateral portal would then be established approximately 1 cm ulnar (posteriorly) along the same line and was used as an alternate working portal from which the lesions could be accurately debrided and the microfractures could be performed. The lesions were debrided with a shaver to a stable bed with removal of all unstable cartilage. Care was taken to preserve and create a circumferential, perpendicular rim of healthy cartilage with a ringed curette. The subchondral bases of the lesions were then picked by microfracture awl to a depth of 2 to 4 mm approximately 3 mm apart beginning at the periphery of the lesion (Figure 1).

The rehabilitation regimen began on the second postoperative day, after drain removal and was divided into 4 sets of 6 weeks. During the first 6 weeks, the focus was mainly on regaining range of motion and contouring the clot caused by the microfractures, first by using a continuous passive motion machine, then after 2 weeks, with active motion assisted by a physiotherapist. If full extension could not be achieved, a nighttime arm-brace fixed in full extension was used. The second set of 6 weeks focused on light strength training, including flexion/extension exercises, pronation/supination exercises, as well as grip exercises. During the third set of 6 weeks, gradually intensifying sport-specific training began, and during the fourth set of 6 weeks, more intensive sport-specific training was allowed.

All patients were brought in for follow-up and were thoroughly examined by an examiner who was not involved in their care. A retrospective review of clinical charts was performed for the date of surgery, preoperative status, radiographic findings, and possible complications. At the final follow-up visit, participants were questioned regarding their level of activity at the time of injury, the time needed for them to return to sports, their level of activity after returning to sports, and any problems resulting from the surgery. Mayo Elbow Performance Indexes (MEPI) were calculated, which were then compared to their pre-operative scores. The MEPI defines excellent results as the scores over 90 points, good scores between 75 and 89, fair scores between 60 and 74, and poor scores under 60 (17).

## Results

The average length of the follow-up was 5.3 years (median 5 years; range 2-9). Success of treatment was determined according to the increase in MEPI on mid-term follow-up and the patients' return to sports. The median MEPI before the operation was 55 (range 15-70). Eight patients scored excellent results on the follow-up and 1 scored a good result (8 MEPI at 100, 1 MEPI at 85). The patient who scored 85 post-operatively reported mild pain very occasionally upon strong exertion of the operated elbow. Four out of 9 patients were able to increase their training intensity, 2 patients returned to the same level of activity, 2 patients changed sports (due to reasons unrelated to the health of their elbow), and 1 patient left professional sports and started training only recreationally (due to decreased ambition). No patients stopped participating in sports altogether (Table 1). There were no perioperative complications and the rehabilitation period was uneventful due to the high compliance of the patients.

## Discussion

Our study showed that 8 patients treated by arthroscopic debridement and microfracture scored excellent results on the follow-up and 1 scored a good result.

There is still much debate in the literature concerning the ideal treatment for OCD of the elbow. The goal of treating OCD of the elbow is to enable adolescent patients to not only perform everyday tasks without pain, locking, or catching, but also to return to their previous level of sporting activity without increasing their risk of developing osteoarthritis (18). Generally accepted guidelines have been proposed for treatment – conservative treatment should be considered in stable lesions – when the affected area is small, still in the early radiolucent stage, range of motion is normal, and the capitellar physis is still open, otherwise results are not favorable (1,6,15,19). Conservative treatment consists mainly of resting the involved elbow, activity modification, anti-inflammatory drugs, and physiotherapy (6,20). Unstable OCD has been defined as having a closed capitellar physis, radiographically nondisplaced or displaced fragments, and restricted range of motion, in which cases surgery is recommended (19).

Surgical treatments are quite varied, and success has been reported to varying degrees with every choice of treatment, though there is general agreement that some form of reconstruction correlates with better outcome than debridement alone (21,22). Surgical treatments can typically be divided into three types: open surgeries that attempt to fix or reattach the loose or free fragment(s), arthroscopic surgeries that involve debridement with or without bone marrow stimulation, and cartilage resurfacing techniques.

Open surgery mainly finds its application when the fragments are large enough and vital enough to warrant fixation. Fragment fixation has been performed with Herbert screw fixation, autologous bone peg grafting, pull-out wiring and bone grafting, and by pinning with dynamic staples (2,23). Excellent results have been obtained in one study using internal fixation with pull-out wiring and bone grafting in 10 out of 11 male baseball players (average age 14.7 years; average follow-up 57 months) with unstable OCD of the capitellum, all of whom returned to their pre-operative level of sports activity (2).

Arthroscopic techniques include debridement, abrasion chondroplasty, absorbable pin insertion, and microfracture (15,24-29). Arthroscopy has also been used to assist in procedures requiring a mini-arthrotomy where drilling is performed for marrow stimulation (30,31). Due to the minimal invasiveness of arthroscopic techniques, scarring is smaller, recovery quicker, and complications less common. Experienced arthroscopists can perform quite versatile operations, having access to the entire elbow joint, and should be able to treat concurrent lesions (Table 2).

**Table 2:** Summary of reports from the literature in which patients underwent arthroscopic surgery due to osteochondritis dissecans (OCD) of the elbow*

First author (year)	Number of patients	Median age (range) at the time of operation in years	Method	Postoperative follow-up in months (range)	Results	Comment
Baumgarten TE (1998) (15)	16†	13.8 (10-17)	debridement, abrasion chondroplasty, and removal of free fragment(s)	48 (24-75)	13 of 16 patients made full return to sports	2 reoperations – one due to missed free fragment, the other due to contracture
Ruch DS (1998)(28)	12	14.5 (8-17)	debridement, and removal of free fragment(s)	39 (24-70)	11 of 12 patients had excellent results, but only 3 returned to sports	1 patient underwent subsequent radial head resection due to continued mechanical symptoms
Byrd JWT (2002)(25)	10	13.8 (11-16)	chondrectomy or abrasion chondroplasty, with removal of free fragments	46.8 (36-72)	4 of 10 patients returned to playing baseball, the rest changed sports	1 reoperation due to contracture, pain, catching, and extension of defect to lateral capitellar rim
Krijnen MR (2003)(26)	5	14.6 (10-19)	debridement, and removal of free fragment(s)	5 (1-6)	2 patients returned to sports	no complications noted, though follow up was relatively short
Brownlow HC (2006)(20)	29	22.0 (11-49)	debridement, and removal of free fragment(s)	77 (7-149)	22 of 27 athletes returned to sporting activities	11 patients had post-operative locking or catching. 2 reoperations: 1 due to posterior impingement, 1 due to osteophyte formation
Bojanić I (2006)(24)	3	14 (13-15)	debridement, microfracture, and removal of free fragment(s)	16 (14-18)	Full return to sport activities in all patients	no complications noted, though follow up was relatively short
Rahusen FT (2006)(27)	15	28 (16-49)	debridement, and removal of free fragment(s)	45 (18-59)	80% returned to sports activities, MAESS score – 65.5 pre-op./ 90.8 post-op	only 2 patients were teenagers
Takeba J (2009)(29)	4	14.5 (12-16)	debridement, removal of free fragment(s), insertion of absorbable pins	6 (3-8)	2 of 4 patients returned to playing baseball so far	microfracture was additionally done in one patient
Jones KJ (2009)(39)	21†	13.1 (10-17)	drilling, removal of free fragment(s)	48 (21-83)	SANE scores were 87 post-op. (range 50-100). 18 patients returned to sports	Only 10 purely arthroscopic drillings. 12 required mini-arthrotomies for bone grafting or removal of large loose bodies.
Schoch B (2010)(40)	13	NA‡	synovectomy, chondroplasty, abrasion arthroplasty, marrow drilling, or loose body removal	43.2 (12-96)	Mean DASH score was 8.6 (0.0-22.41). 4 of 10 patients fully returned to sports	Only 10 patients available for follow-up. 4 surgeons involved in treatment. No preoperative DASH scores.

*Abbreviations: MAESS – Modified Andrews Elbow Scoring System; SANE – Single Assessment Numerical Evaluation; DASH – Disabilities of the arm, shoulder, and hand.

†In one patient, both elbows were operated on.

‡Mean age (range) at presentation: 16 (10-25) years.

Microfracturing, as part of an arthroscopic procedure, is a well established treatment option used to treat osteochondral defects in the knee and ankle – the joints that bear the greatest loads in the human body (10-12,14,32). In addition to the already mentioned benefits of arthroscopy in general, there is a substantially reduced risk of flexion contracture or ectopic ossification, no heat necrosis of surrounding bone (as in drilling), no donor site morbidity (as in mosaicplasty), no delayed joint swelling or bone resorption (as in pinning), and greater cost-effectiveness on the whole (23-25,29,33). Although not a widely practiced method of treating OCD of the elbow, very promising early results encouraged us to pursue this line of treatment further (24). It has been noted in the recent literature that microfracturing has not been shown as beneficial in the mid-term (34,35). Our mid-term results are comparable to those of other authors who have treated OCD of the capitellum with more complex open techniques, which are more successful than performing arthroscopic debridement alone.

Cartilage resurfacing entails either osteochondral autograft transplantation (OAT or mosaicplasty) or autologous chondrocyte implantation (ACI). OATs has been shown to be useful for covering large defects and provide maximum joint surface contact with hyaline cartilage to make a load-bearing joint durable (32). Shimada et al reported excellent clinical and radiographic results in 8 out of 10 patients and poor results in 2 patients (mean follow up 25.5 months) (32). Yamamoto et al reported excellent results in their case series of 18 patients (9 grade 3 and 9 grade 4 on MRI lesions, mean follow-up 3.5 years) (36). Six out of 9 patients with grade 3 lesions and 8 out of 9 patients with grade 4 lesions returned to playing baseball, however, in some cases it took them 2 years to achieve full throwing power. Iwasaki et al reported that 18 of their 19 teenagers had good and excellent mid-term results at their elbows and excellent donor site recovery, with all except two of them returning to their previous level of sports activity (37). Drawbacks of OATs include the procedure's complexity, in which the surgeon must take into account the difference in curvature of the cartilage of the donor site in the knee and the natural curvature of the humeral capitellum (38). Other disadvantages include the occasional excessive and painful bleeding at the donor sites and a mismatch in cartilage thickness of the transplanted plugs and the surrounding capitellar cartilage, potentially causing redistribution in load bearing through the radiocapitellar joint and leading to degeneration of the autograft plugs (37). Autologous chondrocyte implantation has been performed in the elbow in a very limited number of reports. Iwasaki et al reported successful outcomes in 2 patients followed-up for 52 and 57 months, respectively (34).

The main limitation of this study is the small number of patients. This cannot be changed without combining pooled data from multiple centers, since the incidence of OCD of the elbow is rather low. MRI verification of clinical picture was not done on the final follow-up, though it was done in earlier stages (approximately 1 year postoperatively), in which findings of stable defect filling were found. Another limitation is that since this is a relatively new technique as applied to the elbow, not enough time has passed to determine the long term results and there is still a need for prospective trials to test success against other, more commonly done, treatment methods. It should be noted that although by far the most frequent sport associated with OCD of the humeral capitellum is baseball, this sport is far less popular in Croatia than in, for example, the USA and Japan, and so our spectrum of associated sports is significantly different from most publications (2,25,37).

To conclude, our aim was to apply the microfracture technique to treat OCD of the humeral capitellum, which was found to be an effective, minimally invasive technique, and yielded excellent mid-term results in our patients, relieving their pain and allowing them to return to sports within a reasonable amount of time.
